# Biomechanical effects of cement discoplasty on the lumbar spinal unit

**DOI:** 10.3389/fsurg.2022.951141

**Published:** 2022-11-07

**Authors:** Jiajun Huang, Weike Zeng, Ming Li, Ziying Cheng, Junshen Huang, Changchun Liang, Yuxi Li, Lin Huang

**Affiliations:** ^1^ Department of Orthopedics, Sun Yat-sen Memorial Hospital, Sun Yat-Sen University, Guangzhou, China; ^2^ Department of Radiology, Sun Yat-sen Memorial Hospital, Sun Yat-Sen University, Guangzhou, China

**Keywords:** discoplasty, biomechanics, lumbar spine, disc degeneration, orthopedics

## Abstract

**Background:**

Percutaneous cement discoplasty (PCD) is used to treat patients with low back and leg pain due to the intervertebral disc vacuum phenomena. Whether PCD can restore lumbar spinal stability remains unknown.

**Objective:**

The purpose of our *in vitro* study was to evaluate the biomechanical changes brought about by PCD.

**Methods:**

Eight fresh pig lumbar spines were tested in the following order: intact, after nucleotomy, and after discoplasty. Flexion/extension, lateral bending, and axial rotation were induced by pure moments. The range of motion and neutral zone were recorded. A CT scan was performed to assess the injection volume of the bone cement and to observe whether the bone cement was fractured. After removing the facet joint, a compression failure test was conducted to observe the fracture of bone cement.

**Results:**

Compared with nucleotomy, range of motion (ROM) after discoplasty was reduced only in lateral flexion (*P *< 0.05). The results of the neutral zone showed that the neutral zones in flexion–extension and lateral bending were significantly reduced after discoplasty (*P* < 0.05). The neutral zone was more sensitive to changes in lumbar stability than ROM. Bone cement slides were observed during the biomechanical test. The CT scan and compression failure test showed that bone cement fracture was more likely to occur at the puncture channel in the annulus fibrosus region.

**Conclusion:**

In all, the biomechanical study indicates that discoplasty helps enhance the stability of the lumbar spine in flexion–extension and lateral bending, which explains how PCD works for low back pain. Fractures and sliding of bone cement were observed after discoplasty, and this was more likely to occur at the puncture channel in the annulus fibrosus region. This suggests that bone cement displacement after PCD may cause nerve compression.

## Introduction

Low back pain is a common symptom and the leading cause of disability globally (1). Intervertebral disc degeneration is a major cause of low back pain (2). Intervertebral disc vacuum phenomena (VP), referring to a translucent radiographic appearance due to the presence of gas in the lumbar disc region, are one of the characteristics of intervertebral disc degeneration. Intradiscal VP is a common finding observed from spinal radiographs in 20% of elderly patients. Intradiscal VP is usually asymptomatic, but some researchers suggest that intradiscal VP is the cause of dynamic foraminal stenosis and axial instability, which lead to mechanical low back pain and leg pain (3–5). Percutaneous cement discoplasty (PCD) is a minimally invasive technique developed by Varga et al. used to treat patients with low back and leg pain due to intervertebral disc VP (6). The principle of this operation is to inject polymethylmethacrylate (PMMA) into the intervertebral disc with vacuum phenomena to restore stability of the lumbar spine and restore foraminal height (7). At present, studies including a small number of cases and lacking long-term follow-up have shown that PCD can effectively relieve low back and leg pain. The ability of discoplasty to restore the height of the intervertebral space and improve the physiological curvature of the lumbar spine was also demonstrated by radiology (8, 9). However, whether PCD can restore lumbar spinal stability remains unknown. It is important to determine whether PCD can improve lumbar stability for the clinical application of PCD, especially in determining whether PCD can be used in treating mechanical low back pain. The purpose of our *in vitro* study was to evaluate the biomechanical changes brought about by PCD using a standardized biomechanical protocol.

## Materials and methods

### Specimen preparation

**Figure 1:**
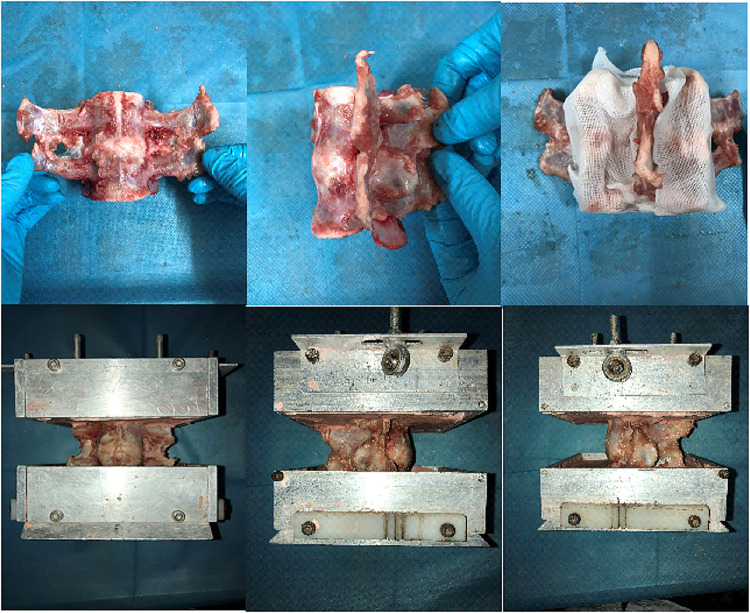
Specimen pretreatment and embedding.

Eight fresh-frozen pig specimens (range 10–12 months old), consisting of L5–L6, were used in this study. The specimens were obtained from food markers in Guangzhou. After examining the specimens, CT scans were taken to exclude fractures and deformities. Specimens were obtained fresh-frozen at −20 °C and then thawed in a bath of normal saline at 25 °C before the experiment. The average time between slaughter and experimentation was 3 days. The paravertebral musculature was removed, while all ligaments, joints, and discs were preserved. Short screws were partially driven into the L5 and L6 vertebrae to anchor vertebrae in the plaster powder better. Then, the L5 and L6 vertebral bodies were embedded in the plaster (Figure 1). Two Kirschner wires with Plexiglas markers at the end were driven horizontally into the L5 and L6 vertebral bodies so that the mark and the vertebral body movement were coordinated and the motion of the specimens was tracked by using a motion analysis system (Optotrak Certus System; Northern Digital Inc., Waterloo, Canada). The locations of the marker, which denoted a rigid body, were aligned sagittally along the curvature of the spine. To investigate the biomechanical effect of PCD on the lumbar spine, after an intact condition test, the nucleotomy model of the L5–L6 segment was prepared to simulate degenerated discs according to the method described below. The specimens were treated with discoplasty after being tested in degenerated conditions. During preparation and testing, the specimens were kept moist with 0.9% saline solution to prevent dehydration. All tests were carried out at room temperature, 27 °C. Upon completion of these tests, a CT scan of the specimen was carried out to observe the distribution of bone cement.

### Nucleotomy

**Figure 2:**
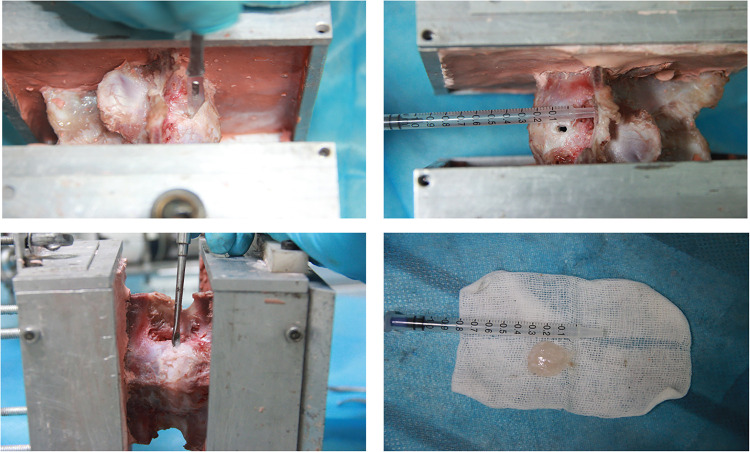
Construction of the nucleotomy condition.

To simulate the intradiscal VP, a 5-mm horizontal incision was made on the left side of the annulus fibrosus region of the specimen, through which the nucleus pulposus was scraped with a curette (Figure 2) (10).

### Discoplasty

**Figure 3:**
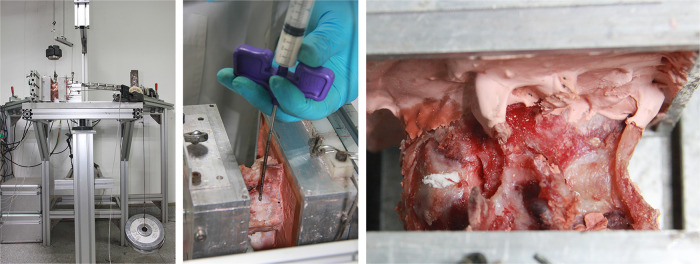
Construction of the discoplasty condition.

To maintain the intervertebral disc space after the resection of the nucleus pulposus, we applied axial traction during the injection of bone cement. High-viscosity radiopaque acrylic bone cement (10% BaSO4) (Wego, China) was injected inside the disc through the incision. After injection, the cement was hardened for 30 min (Figure 3).

### Mechanical testing

**Figure 4:**
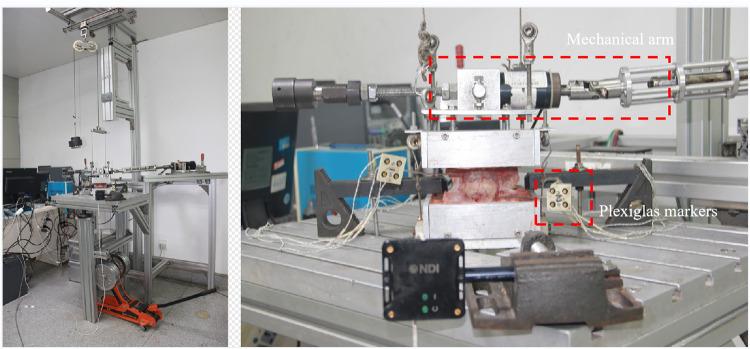
Composition of the mechanical test system and installation of specimens.

The test method complies with the testing criteria for spinal implants (11). The composition of the mechanical test system and installation of specimens are shown in Figure 4. A continuous pure moment of ±7.5 Nm was applied to the specimen in flexion–extension (FE), lateral bending (LB), and axial rotation (AR), with a compressive follower preload of 300 N at room temperature on a spinal simulator based on the principles of Crawford et al. (12) The load cycle consists of a displacement-controlled loading phase with a velocity of 1°/s due to the laxity of the specimen followed by a load-controlled phase starting at 2% below the maximum load (7.5 Nm), which was kept constant for 1 s once the maximum load (7.5 Nm) was reached. The six components of motion were obtained from the Optotrak Certus data files in the form of Euler angles (°). The range of motion (ROM) and neutral zone (NZ) of the characteristic parameters were analyzed from the hysteresis curves of the third loading cycle, according to Wilke et al.

### Compression failure test

The facet joints of the specimen were removed, and the specimen was placed in an electronic universal testing machine for the compression failure test (loading speed 1 mm/min, maximum load 10,000 N). Stress attenuation of 80% or beyond the measurement range was taken as the standard at the end of the experiment.

### Data analysis

Because of the limited sample size, the Kruskal–Wallis test was conducted for the ROM and neutral regions under different conditions. The pairwise comparison was tested by Dunn's multiple-test method. All statistical analyses were processed using SPSS for Windows, version 20.0 (SPSS, Chicago, IL, USA). A *P* value of <0.05 was considered significant.

### Ethics

The samples used in this study were taken from pigs killed for food in abattoirs, so the IRB/ethics committee approval was not required for this study.

## Results

### Range of motion

**Figure 5:**
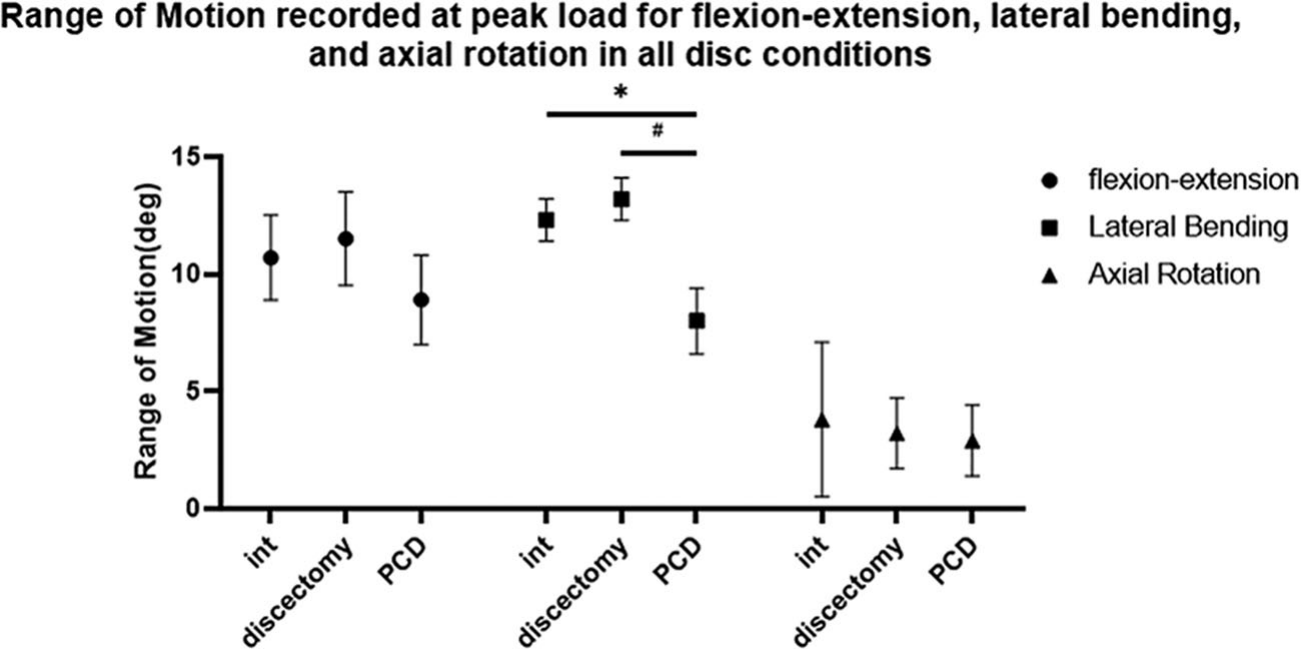
ROM values for different conditions (*n* = 8, ±SD, degree, °). * *P* < 0.05: compared with the Int group; ^#^*P* < 0.05: compared with the nucleotomy group.

The range of motion of the specimens under various conditions is shown in Table 1 and Figure 5. The ROM of the specimens was not significantly different between nucleotomy and intact conditions. The ROM in lateral bending significantly increased after discoplasty compared with the nucleotomy condition (*P* < 0.05). ROM was not significantly different between nucleotomy and discoplasty in flexion–extension (*P* = 0.730) and axial rotation (*P* = 1.000).

**Table 1:** ROM values for different conditions (*n* = 8, ±SD, degree, °).

Specimen	Flexion–extension	Lateral bending	Axial rotation
Int	10.7 (1.8)	12.3 (0.9)	3.8 (3.3)
Nucleotomy	11.5 (2.0)	13.2 (0.9)	3.2 (1.5)
PCD	8.9 (1.9)	8.0 (1.4)*,^#^	2.9 (1.5)

ROM, range of motion; PCD, percutaneous cement discoplasty.

* *P* < 0.05: compared with the Int group.

^#^ *P* < 0.05: Compared with the nucleotomy group.

### Neutral zone

**Figure 6:**
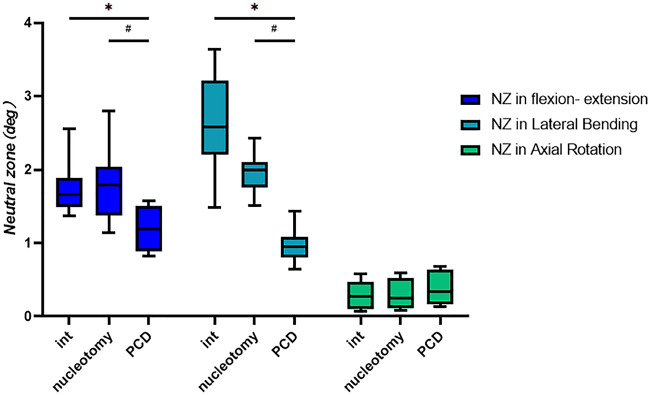
Neutral zones in different conditions. (n = 8, ±SD, degree, °). *P < 0.05: compared with the Int group; ^#^*P* < 0.05: compared with the nucleotomy group.

The neutral zones of the specimens under various conditions are shown in Table 2 and Figure 6. Nucleotomy decreased the NZ in lateral bending (*P* < 0.05). However, there was no statistically significant difference in the NZ in flexion–extension and axial rotation between the nucleotomy and intact conditions. Discoplasty reduced the NZ in flexion–extension and lateral bending compared to the intact and nucleotomy conditions (*P *< 0.05). In the NZ in axial rotation, there was no significant difference between the three conditions.

**Table 2:** Neutral zones in different conditions (*n* = 8, ±SD, degree, °).

Speciment	NZ in flexion–extension	NZ in lateral bending	NZ in axial rotation
Int	1.8 (0.4)	2.6 (0.7)	0.3 (0.2)
Nucleotomy	1.8 (0.5)	2.0 (0.3)*	0.3 (0.2)
PCD	1.2 (0.3)*,^#^	1.0 (0.2)*,^#^	0.4 (0.2)

NZ, neutral zone; PCD, percutaneous cement discoplasty. *P < 0.05: Compared with the Int group.

^#^ *P* < 0.05: compared with the nucleotomy group.

### CT scan of bone cement

**Figure 7:**
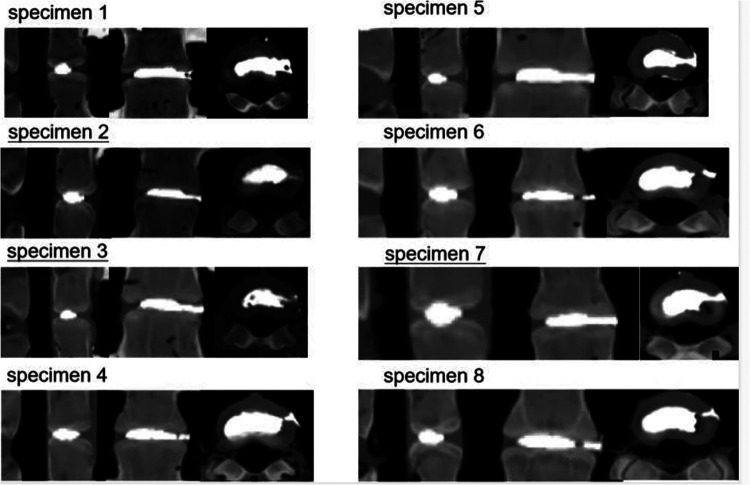
CT scan of specimens treated with discoplasty.

After completing mechanical testing, we performed a CT scan on the specimen to assess the injection volume of the bone cement and to observe whether the bone cement was fractured. The volume of bone cement injection was 1.38 ± 0.23 cm^3^. Bone cement was evenly distributed within the intervertebral disc, and three specimens showed a partial fracture of the bone cement in the puncture channel of the annulus fibrous region (Table 3 and Figure 7).

**Table 3:** CT scan of specimens treated with discoplasty.

Specimen	1	2	3	4	5	6	7	8	Mean (±SD)
Cement volume (cm^3^)	1.34	1.35	1.15	1.86	1.1	1.41	1.32	1.51	1.38 (±0.23)
Cement surface area (mm^2^)	301	279.8	282.3	402.1	257.9	316	305.7	307.6	306.55 (±42.97)
Cranial endplate area (mm^2^)	740.3	714.7	733.4	821.3	727.7	691.8	701.7	765.4	737.04 (±42.07)
Caudal endplate area (mm^2^)	613.1	632.5	655.1	692.8	614.3	632.7	658.1	646.5	643.14 (26.19)
PMMA crack within the annules	−	−	−	+	−	+	−	+	

PMMA, polymethylmethacrylate.

### Displacement of bone cement

During mechanical testing in the discoplasty condition, we observed sliding of bone cement, especially in lateral bending. The sliding range of bone cement at the puncture entrance was 1–3 mm [Supplementary materials (gif S1)]. Considering that the CT scan showed a bone cement fracture at the puncture site of some specimens, we believed that bone cement had a risk of fracture and displacement. To observe whether the bone cement has structural weakness after discoplasty, a compression failure test was carried out on the specimen.

### Compression failure test

**Figure 8:**
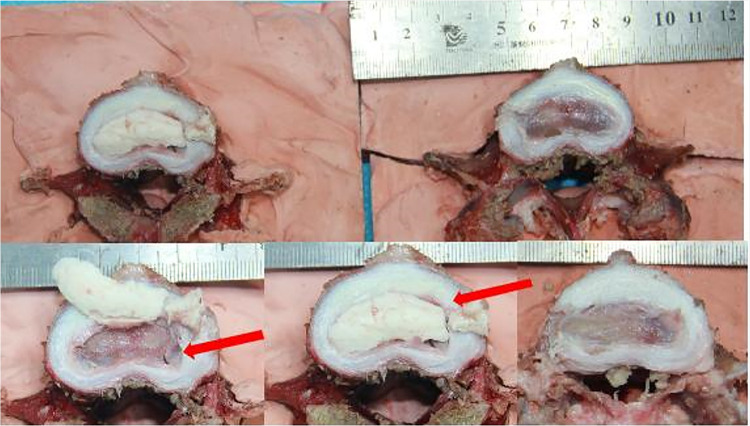
Structural changes of specimens subjected to compression failure tests. The red arrow indicates the fracture site of the vertebral body and bone cement.

The results of the compression failure test are shown in Table 4 and Figure 7. Three specimens did not reach yield strength due to range limitations. The yield strength of the other five specimens ranged from 8.86 to 9.47 kN. Among these five specimens, there was no fracture of bone cement in the nucleus pulposus area, three specimens showed a fracture of the cranial endplate, and one specimen showed a fracture of the caudal endplate (Figure 8). Of all the specimens, we observed an increase of three cases of cement fracture in the annulus fibrous puncture channel.

**Table 4:** Structural changes of specimens subjected to compression failure tests.

Specimen	1	2	3	4	5	6	7	8
Cranial endplate fracture	+	+	+	−	+	−	−	−
Caudal endplate fracture	+	−	−	−	−	−	−	−
PMMA crack within the nucleus puposus region	−	−	−	−	−	−	−	−
PMMA crack within the annules fibrosus region	−	+	+	+	−	+	+	+
Force of structural failure (kN)	8.8583	9.4691	9.0496	−	9.0499	−	9.2044	−

PMMA, polymethylmethacrylate.

## Discussion

Percutaneous cement discoplasty, as a minimally invasive spinal surgery, is a promising treatment option for elderly patients with severe low back pain and/or leg pain due to the intervertebral vacuum phenomenon, especially those who cannot tolerate open spinal surgery. The intervertebral vacuum phenomenon causes vertical instability of the lumbar spine and dynamic stenosis of the intervertebral foramen, causing low back pain and leg symptoms. Cement discoplasty has a good immediate stabilizing effect and restores and maintains foraminal height. Kiss et al. found that foraminal height increased in patients after discoplasty; however, whether discoplasty can restore lumbar stability and maintain foraminal height remains unknown.

In this study, we analyzed the ROM and neutral zone of lumbar specimens in intact, nucleotomy, and discoplasty conditions to explore the effect of discoplasty on lumbar spine stability. Compared with after nucleotomy condition, ROM after discoplasty was reduced only in lateral flexion. In another biomechanical study of discoplasty, there was no decrease in the ROM of the specimen after discoplasty (10). Previous studies have suggested that the intervertebral disc vacuum sign can reduce the sagittal stability of the lumbar spine and lead to mechanical low back pain in patients. According to the ROM values, discoplasty had little effect on the sagittal stability of the lumbar spine. This is obviously not consistent with clinical studies that have reported the benefits of discoplasty in reducing low back pain. Considering the parameters of spinal stability, the neutral zone was found to be a more sensitive parameter than the range of motion (13, 14). Therefore, we further analyzed the changes in the neutral zone of the specimens. The results of the neutral zone showed that the neutral zones in flexion–extension and lateral bending were significantly reduced after discoplasty. This suggests that discoplasty does have a role in restoring stability to the lumbar spine. In axial rotation, the main load is transmitted over the facet joints (15). It is therefore understandable that no significant changes in the ROM or neutral zone in axial rotation were observed after discoplasty.

During the mechanical testing of specimens in discoplasty conditions, we observed displacement of bone cement, especially in lateral bending. The sliding range of bone cement at the puncture entrance was 1–3 mm. The annulus fibrosus region of the specimen is relatively intact except for a puncture opening on the left. In patients undergoing discoplasty, the annulus fibrosus region may have varying degrees of cleft due to severe degeneration. At the same time, the removal of the paravertebral musculature also made it difficult to accurately reflect the possible sliding distance and direction of bone cement through this experiment. Follow-up observation of postoperative cement displacement in patients undergoing discoplasty is necessary.

In the postoperative x-ray and CT images provided in the literature published by Varga et al., we found that bone cement fracture occurred in some patients after receiving treatment with PCD, which is consistent with the situation of bone cement fracture in some specimens after intervertebral disc molding in our study. We further confirmed that the bone cement at the puncture channel was a structural weak point through compression failure tests and anatomical observations of the specimens. Sola et al. suggested that transpedicular S1 access was more convenient to reach the disc space than extrapedicular access for the L5–S1 level to avoid the influence of the L5 transverse process and iliac spine on puncture. We believe that applying this approach to other levels may help reduce the risk of cement fracture and displacement.

In conclusion, the biomechanical study indicates that discoplasty helps enhance the stability of the lumbar spine in flexion–extension and lateral bending but not in axial rotation. Fracture and sliding of bone cement were observed after discoplasty, which was more likely to occur at the puncture channel in the annulus fibrosus region. The fracture and sliding of bone cement after discoplasty are alarming. Further clinical studies are needed to confirm the surgical outcome and complications of discoplasty.

## Data Availability

The original contributions presented in the study are included in the article/**Supplementary Material**, further inquiries can be directed to the corresponding author.
